# Association between the Polymorphisms of *fads2a* and *fads2b* and Poly-Unsaturated Fatty Acids in Common Carp (*Cyprinus carpio*)

**DOI:** 10.3390/ani11061780

**Published:** 2021-06-15

**Authors:** Yan Zhang, Xiao-Qing Sun, Yu-Qing Ye, Qi Wang, Qing-Song Li, Ran Zhao, Hong-Wei Wang, Jiong-Tang Li

**Affiliations:** 1 Key Laboratory of Aquatic Genomics, Ministry of Agriculture and Rural Affairs, and Beijing Key Laboratory of Fishery Biotechnology, Chinese Academy of Fishery Sciences, Beijing 100141, China; zhangy@cafs.ac.cn (Y.Z.); sunxiaoqing@cafs.ac.cn (X.-Q.S.); wangqi@cafs.ac.cn (Q.W.); zhaoran@cafs.ac.cn (R.Z.); wanghongwei@cafs.ac.cn (H.-W.W.); 2 National Demonstration Center for Experimental Fisheries Science Education, Shanghai Ocean University, Shanghai 201306, China; yeyuqing95@163.com (Y.-Q.Y.); liqingsong686@163.com (Q.-S.L.)

**Keywords:** common carp, poly-unsaturated fatty acid, coding SNP, fatty acid desaturase

## Abstract

**Simple Summary:**

Fishes are the major dietary source of polyunsaturated fatty acids (PUFAs) for humans. The limited availability of PUFAs derived from fish represents a critical bottleneck in food production systems, one that numerous research institutions and aqua-feed companies in this field are trying to overcome. This problem could be minimized by select-bred fish to be capable of more effectively producing endogenous PUFAs. Fatty acid desaturase 2 (*fads2*) is one of the rate-limiting enzymes in the synthesis of PUFAs. The common carp, one of the most important food and ornamental allo-tetraploid fish, encodes two *fads2* genes (*fads2a* and *fads2b*). The PUFA contents among common carp individuals were numerous, suggesting that there might exist polymorphisms in *fads2a* and *fads2b*. However, selective breeding of common carp with high PUFA contents was hindered due to a lack of effective molecular markers. This study investigated the contents of PUFAs in common carp and identified polymorphisms in the CDS regions of *fads2a* and *fads2b*. The association study identified three cSNPs associated with the PUFA contents and suggested that *fads2b* might be the major gene responding for common carp PUFA contents. These cSNPs would be potential markers for future selection to improve the PUFA contents in common carp.

**Abstract:**

Fatty acid desaturase 2 (*fads2*) is one of the rate-limiting enzymes in PUFAs biosynthesis. Compared with the diploid fish encoding one *fads2*, the allo-tetraploid common carp, one most important food fish, encodes two fads2 genes (*fads2a* and *fads2b*). The associations between the contents of different PUFAs and the polymorphisms of *fads2a* and *fads2b* have not been studied. The contents of 12 PUFAs in common carp individuals were measured, and the polymorphisms in the coding sequences of *fads2a* and *fads2b* were screened. We identified five coding single nucleotide polymorphisms (cSNPs) in *fads2a* and eleven cSNPs in *fads2b*. Using the mixed linear model and analysis of variance, a synonymous *fads2a* cSNP was significantly associated with the content of C20:3n-6. One non-synonymous *fads2b* cSNP (fads2b.751) and one synonymous *fads2b* cSNP (fads2b.1197) were associated with the contents of seven PUFAs and the contents of six PUFAs, respectively. The heterozygous genotypes in both loci were associated with higher contents than the homozygous genotypes. The fads2b.751 genotype explained more phenotype variation than the fads2b.1197 genotype. These two SNPs were distributed in one haplotype block and associated with the contents of five common PUFAs. These results suggested that *fads2b* might be the major gene responding to common carp PUFA contents and that fads.751 might be the main effect SNP. These cSNPs would be potential markers for future selection to improve the PUFA contents in common carp.

## 1. Introduction

Fish provide polyunsaturated fatty acids (PUFAs) for human. PUFAs are necessary for important biological functions in human, such as regulating lipid metabolism, stimulating growth and development, exerting anticancer and antiaging effects, enforcing immune-regulation, promoting cardiovascular health, and aiding weight loss [1,2,3]. PUFAs in the diet also help to elevate serum peroxides and antioxidant reserves and to lower plasma triglycerides, thus helping to ameliorate cardiovascular diseases [4].

Biosynthesis of PUFAs in fish involves desaturation and elongation of the precursor C18 FA [5,6]. The fatty acid desaturase 2 (*fads2*), an endoplasmic reticulum membrane-bound enzyme, introduces *cis* double bonds into fatty acyl chains at specific sites of PUFAs [7,8,9]. This gene is one of the rate-limiting enzymes in the PUFA biosynthesis process. Although most diploid fish encode only one *fads2* gene, comparative analysis revealed that mutations in the protein sequences affect the substrate specificity (Δ4, Δ5, Δ6, and Δ8 activities) and conversion efficiency [10,11,12,13,14], leading to different types and contents of PUFAs among fish. Furthermore, single nucleotide polymorphisms (SNPs) of *fads2* in human, mammals, and fish [15,16,17,18,19,20] are associated with omega-6 (n-6) and omega-3 (n-3) FA levels. Therefore, identifying mutants among fishes or polymorphisms among varieties in one fish would benefit the future breeding selection of fish having high contents of PUFAs. 

The common carp (*Cyprinus carpio*) is cultured commercially worldwide, generating numerous geographical populations. Domestication has also accelerated the phenotypic diversity of the common carp [21]. This fish is able to convert dietary C18 FAs to PUFAs[6,11]. Due to its allo-tetraploiy [22], the common carp encodes one more *fads2* gene than most diploid freshwater fish. Two fad cDNAs, *fads2a* and *fads2b*, were previously identified [23]. The PUFA contents among common carp individuals were numerous [24], suggesting that there might exist polymorphisms in *fads2a* and *fads2b*. Indeed, in common carp var. Jian, one intronic SNP in *fads2a* and one intronic SNP in *fads2b* were identified [23]. However, whether these two intronic SNPs were associated with the PUFA contents has not been studied. The question of whether there are polymorphisms in these two genes in other varieties and the association between polymorphisms and the PUFA contents also require studying. Furthermore, which of these two genes is the major gene responsible for PUFA biosynthesis is still unknown.

Genomic markers have been demonstrated to be efficient in the representation of the associated phenotypes and aquaculture breeding [25,26,27]. Selective breeding of common carp with high PUFA contents is hindered due to a lack of effective molecular markers. To answer the above questions and to develop SNP markers for selective breeding of PUFAs, in this study, we measure the PUFA contents and identified more polymorphisms in common carp *fads2a* and *fads2b* than before. The association between certain genotypes and the contents of multiple PUFAs in the common carp population are performed. Three significantly associated SNPs are useful in the molecular-assisted selection of high PUFA contents in common carp. 

## 2. Materials and Methods

### 2.1. Ethics Statement

This study was conducted following the recommendations of the Care and Use of Animals for Scientific Purposes established by the Animal Care and Use Committee of the Chinese Academy of Fishery Sciences (ACUC-CAFS). Before collecting the tissues, all fishes were euthanized in MS222 solution.

### 2.2. Sampling

To increase the diversities of HUFA contents, in 2018, we collected the juvenile common carp including the var. Huanghe (HHC, at Donge, Shandong province, China), the var. Furui (FRC), and the var. Jian (JC, at Wuxi, Jiangsu province, China) (Supplementary Figure S1). The samples were maintained at the experimental fish farm of Chinese Academy of Fishery Sciences (Fangshan, Beijing, China) and fed with the same commercial diet (Tongwei, China) in the culture ponds. This commercial diet contained fish meal, fish oil, colza oil, and soybean meal. In 2019, a total of 269 one-year-old common carp were randomly selected, including 124 FRC individuals, 47 HHC individuals, and 98 JC individuals. The liver and muscle of each individual were dissected, immediately frozen in liquid nitrogen, and stored at −80 °C. 

### 2.3. RNA Extraction, cDNA Synthesis, Sequencing, and Genotyping

RNA was extracted from the liver of each individual using TRIzol® (100 mg sample /1 mL TRIzol, Invitrogen, Waltham, MA, USA). The concentration of RNA was determined using the NanoDrop 2000 spectrophotometer (Thermo Scientific™, Wilmington, DE, USA), and its quality was determined by 1% agarose gel electrophoresis. Next, 1 µg RNA was reverse transcribed to cDNA using the SuperScript III RNase H-Reverse Transcriptase Kit (Invitrogen, Carlsbad, CA, USA) with oligo-dT. Based on the reference full-length sequence of *fads2a* and *fads2b* in common carp (GenBank accessions: MK852165 and MK852166), gene-specific primers were designed to amplify the complete coding sequence (CDS) regions of these two genes (Table 1). 

PCR amplifications were performed in a total volume of 15 μL, containing 50 ng of cDNA, 1.5 mM of MgCl_2_, 0.2 mM of each dNTP, 15 pmol of each primer, and 0.3 U of Taq polymerase (TransGen Biotech, Beijing, China). The following thermal profile was applied: 5 min at 94 °C, 40 cycles of 30 s at 94 °C, 30 s at the annealing temperature of 56/62 °C (Table 1), 1 min at 72 °C, and a final extension of 10 min at 72 °C. Finally, the PCR products were sequenced with the Sanger method. Since the CDS of each gene was less than 1350 bp, two ends of one round of Sanger sequencing covers the complete CDS. 

Since *fads2a* and *fad2b* have high nucleotide sequence similarities at 89.86%, to avoid misalignments and putative artificial coding SNPs (cSNPs), all of the amplified sequences were aligned to *fads2a* and *fads2b* using blastn. For one sequence expected to be from *fads2a*, if it had a higher alignment score for *fads2b* than *fads2a*, then this sequence should be aligned to *fads2b* to call for SNPs. The same operation was performed on all sequences expected from *fads2b*. To detect SNPs and to perform genotyping in each sample, the sequences were aligned to their corresponding reference genes in the novoSNP software [28] with the position ranging from 100 bp to 700 bp. If one locus had only one peak, this locus was considered a homozygote. If the base in this locus was different from the reference, then this locus was a cSNP. For one locus with two peaks, the genotype was auto-detected with the software and considered a heterozygous site. If one site had an F-score over 30 measured by novoSNP, it was of high quality. 

### 2.4. Genetic Diversities of Common Carp fads2a and fads2b

We investigated the genetic distances and structures among three varieties with all retained genotypes in *fads2a* and *fads2b*. The genetic distances were estimated using Principle Component analysis (PCA) in the Tassel 5.0 software [29]. The first two eigenvectors were plotted. The population structure among three varieties we analyzed using Admixture 1.3.0 [30]. K was set from 2 to 6. The population structures and the genetic compositions of each individual were visualized by pophelper v2.3.1 [31]. 

As three varieties were grouped together in the PCA analysis and had similar structures, we combined these varieties into one population. The observed heterozygosity (Ho), expected heterozygosity (He), genotype frequency, and allele frequency in the population were calculated using the Genepop software [32]. If the frequency of one SNP in the population was higher than 0.04 (at least 5 out of 122 individuals with *fads2a* genotype information), it was retained in the downstream analysis. The polymorphism information content (PIC) for each retained SNP locus was estimated using PICcalc 0.6 [33]. The effects of SNPs to the coding sequences were classified into synonymous substitution, non-synonymous substitution, stop gain, and stop loss. 

### 2.5. Measuring the Contents of 12 PUFAs in Common Carp

FAs in each individual muscle were transformed into fatty acid methyl esters (FAMEs). FAMEs were prepared, extracted, and purified by thin-layer chromatography following Li et al.’s strategy [34]. The type of each FA was identified using the 7890A GC System (Agilent Technologies, Wilmington, DE, USA) equipped with a flame ionization detector. Samples were subjected to a temperature gradient from 60 °C to 150 °C at 10 °C/min, then to 200 °C at 15 °C/min, and finally to 230 °C at 30 °C/min. Each FAME was identified by comparing its GC retention time with the peaks in a Supelco 37 Component FAMEs standard mix (Nu-chek Prep, Inc., Elysian, USA). We calculated all areas of 25 long FAs (C14:0, C15:0, C16:0, C16:1, C17:0, C17:1, C18:0, C18:1n-9, C18:2n-6, C18:3n-6, C18:3n-3, C18:4n-3, C20:0, C20:1, C20:3n-6, C20:4n-6, C20:3n-3, C20:4n-3, C20:5n-3, C22:0, C23:0, C22:4n-6, C22:5n-6, C22:5n-3, and C24:0). We focused on studying the substrates and the products in the PUFA biosynthesis pathway [34], including nine n-3 PUFAs and nine n-6 PUFAs. Since there was no GC retention time for four PUFAs having at least 24 carbons, we did not detect the contents of these PUFAs. In addition, two PUFAs (C20:2n-6 and C22:6n-3) were not detected in at least half of the samples and were therefore not included in the following analysis. Among the remaining 12 PUFAs (C18:2n-6, C18:3n-6, C18:3n-3, C18:4n-3, C20:3n-6, C20:4n-6, C20:3n-3, C20:4n-3, C20:5n-3, C22:4n-6, C22:5n-6, and C22:5n-3), the proportion of each PUFA was calculated as (area of one PUFA/total area of 25 FAs) × 100. 

### 2.6. PUFA Content Comparison

Before the following association study, we first examine whether there existed significant differences in the PUFA types and contents among three varieties. With the contents of 12 PUFAs, the distances measured by the PCA analysis were estimated with Tassel 5.0. 

The Spearman correlation coefficient of the contents of any two PUFAs in all individuals was calculated using the R ‘cor.test’ function. Using the TBtools software [35], we clustered 12 PUFAs on the basis of the correlations using the ‘Median method’ and visualized all correlation coefficients using the R function ‘heatmap’.

### 2.7. Associations of Polymorphisms in fads2a and fads2b with the Contents of 12 PUFAs

Associations between the contents of each PUFA and the genotypes were studied using the mixed linear model (MLM) and the analysis of variance (ANOVA), respectively. The MLM model, including the PCA and the kinship matrix, which is MLM (PCA + K), was applied into the association study with Tassel 5. This method was widely used in the association study between the polymorphisms in candidate genes and phenotypes [36,37,38]. The percentage of phenotypic variation explained by each marker was calculated with Tassel 5.

For each SNP locus, we grouped the individuals on the basis of their genotypes. Then, we performed pairwise group comparison with ANOVA to estimate whether there existed significant content difference in each PUFA between the two groups. All *p* values were corrected using the false discovery rate (FDR) method for multiple hypothesis testing.

As for each PUFA, one SNP locus was considered significantly associated with the content if it had a *p* value < 0.05 in the MLM method and a FDR-corrected *p* value < 0.05 in the ANOVA. In each gene, the pairwise linkage disequilibrium (LD) between any two SNPs was measured by D’ calculated with PLINK v1.90 [39] and visualized with the R package LDheatmap [40]. The haplotype block was defined if the average D’ of any two compared SNPs in this region was greater than 0.6. 

## 3. Results

### 3.1. Genotyping Common Carp fads2a and fads2b 

The CDS sequences of *fads2a* and *fads2b* were highly similar, with a nucleotide identity of 89.86% and a protein identity of 92.06%. The *fads2a* CDS spanned 12 exons, and the *fads2b* CDS covered 12 exons. For *fads2a*, base contents of A, G, C, and T in the sequence were 25.0%, 26.2%, 24.6%, and 24.2%, respectively; the A + T content (49.2%) was lower than the G + C content (50.8%). For *fads2b*, the average A, G, C, and T base contents in amplified sequences were 25.4%, 26.1%, 24.5%, and 24%, respectively; the A + T content (49.4%) was lower than the G + C content (50.6%). All of these data suggest high similarity between the CDS of these two genes. After amplification and validating the real source genes, we successfully sequenced the entire CDS of *fads2a* in 122 individuals and the entire CDS of *fads2b* in 237 individuals. 

We obtained ten *fads2a* cSNPs and 27 *fads2b* cSNPs (Supplementary Table S1). After filtering low-frequency cSNPs with a MAF threshold of 0.04, five cSNPs, including one non-synonymous SNPs and four synonymous SNPs, were identified in *fads2a* from 122 samples, corresponding to 14 genotypes (Table 2). These cSNPs were distributed in four exons. Likewise, eleven cSNPs, including six non-synonymous SNPs and five synonymous SNPs were identified in *fads2b* from 237 samples (Table 2), being distributed over exons 1 to 11. In these loci, 30 genotypes were detected. Taking the CDS lengths into consideration, the SNP loci only accounted for 0.37% in *fads2a* and 0.82% in *fads2b*, suggesting low polymorphism in these two genes. However, *fads2b* had higher polymorphisms than *fads2a*. 

### 3.2. Genetic Diversities of fads2a and fads2b

**Figure 1:**
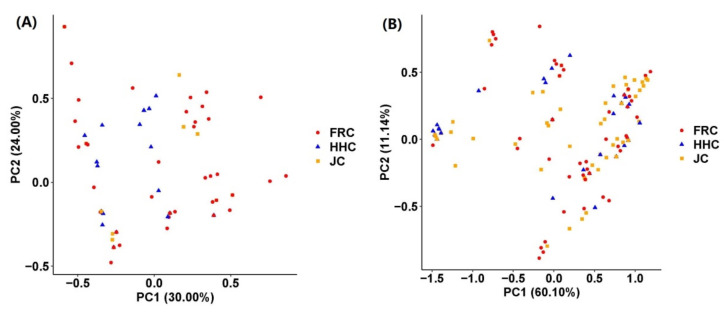
The PCA plots clustering three common carp varieties with the genotypes of *fads2a* (**A**) and *fads2b* (**B**).

PCA based on the *fads2a* genotypes clustered three common carp varieties together (Figure 1A). The first two principal components explained 30% and 24% of the total genetic variances. A similar phenomenon of clustering together was observed with the *fads2b* genotypes (Figure 1B). The first two principal components explained 60.1% and 11.14% of the total genetic variances, respectively. These data proved that these three varieties had similarity genetic background in both *fads2a* and *fads2b.* These findings were further supported by the population structure analysis with *fads2a* cSNPs and *fads2b* cSNPs, respectively (Supplementary Figure S2). In the admixture plots with different K values, three varieties had similar genetic components. There was no variety-specific SNP in these two genes, as evidenced by both PCA and population structure analysis. Therefore, we combined these three varieties into one population in the following analysis. 

The minor allele frequencies (MAF) of *fads2a* cSNPs ranged from 0.05 to 0.28. The MAFs of *fads2b* cSNPs ranged from 0.04 to 0.47. No significant MAF distribution differences between these two genes were observed (Mann–Whitney U test, *p* value = 0.377). Ho in *fads2a* ranged from 0.1 to 0.51 and He ranged from 0.09 to 0.4. PIC analysis indicated that the fads2a.288 locus displayed a low polymorphism level (PIC < 0.1) compared with the other four loci. Among the *fads2b* cSNPs, Ho ranged from 0.02 to 0.38 and He ranged from 0.07 to 0.49. Two loci displayed low polymorphism levels (PIC < 0.1) compared with the remaining nine loci. All *fads2a* cSNPs and *fads2b* cSNPs significantly deviated from the Hardy–Weinberg equilibrium (Table 2, chi-square *p* value < 0.05).

### 3.3. Diversities of PUFA Contents in Common Carp 

We obtained the contents of 12 PUFAs in 269 common carp samples (Supplementary Table S2). PCA analysis with the contents of 12 PUFAs demonstrated that there were no variety-enriched PUFAs (Supplementary Figure S3), similar to no variety-specific SNPs in these two genes. Hence, in the following phenotype comparison and association study, we also grouped three varieties into one population. The n-6 PUFA contents (44.68 ± 16.81%) were higher than the n-3 PUFA contents (6.76 ± 3.58%). The most abundant PUFA was C18:2n-6 (24.33 ± 11.03%), followed by C20:4n-6 (8.17 ± 4.96%) and C18:3n-6 (6.36 ± 6.75%) (Table 3). The content distributions of 12 PUFAs were not subject to the normal distribution (Kolmogorov–Smirnov test *p* value < 0.05, Supplementary Figure S4). 

**Figure 2:**
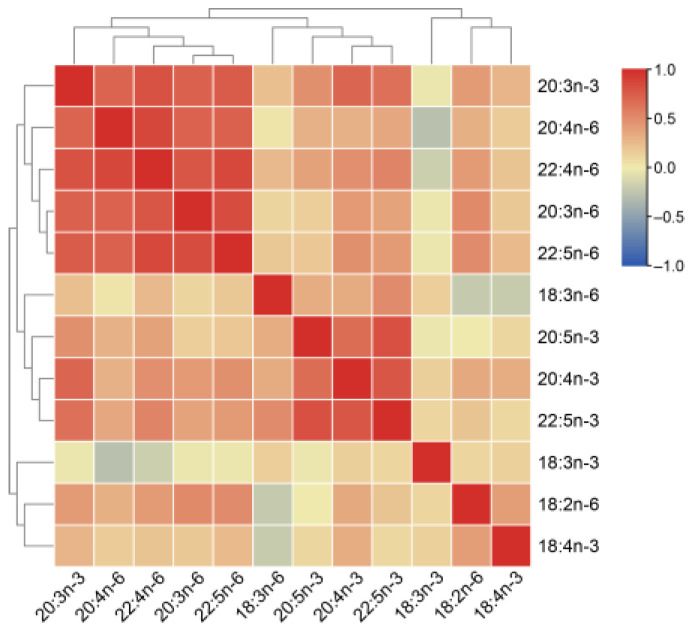
Heat map shows content correlations among 12 PUFAs.

A correlation matrix of pairwise content comparisons between two PUFAs (Figure 2) exhibited two major clades. The first clade including PUFAs with 18 carbons. The components in the second clade mainly included at least 20 carbons (except C18:3n-6), which were the derivates of the first clade. Hence, it is reasonable that there exist negative correlations between these two clades. Based on the correlations among the components, the second clade was further classified into two groups: n-6 PUFAs (except C20:3n-3) and n-3 PUFAs. The second-clade classification was consistent with the classification based on the location of the last double bond with reference to the terminal methyl end of the molecule (n-3 and n-6) [11]. The clustering suggested that correlations among the PUFA products were higher than those between the products and substrates. 

### 3.4. Three cSNPs Associated with the PUFA Contents 

**Figure 3:**
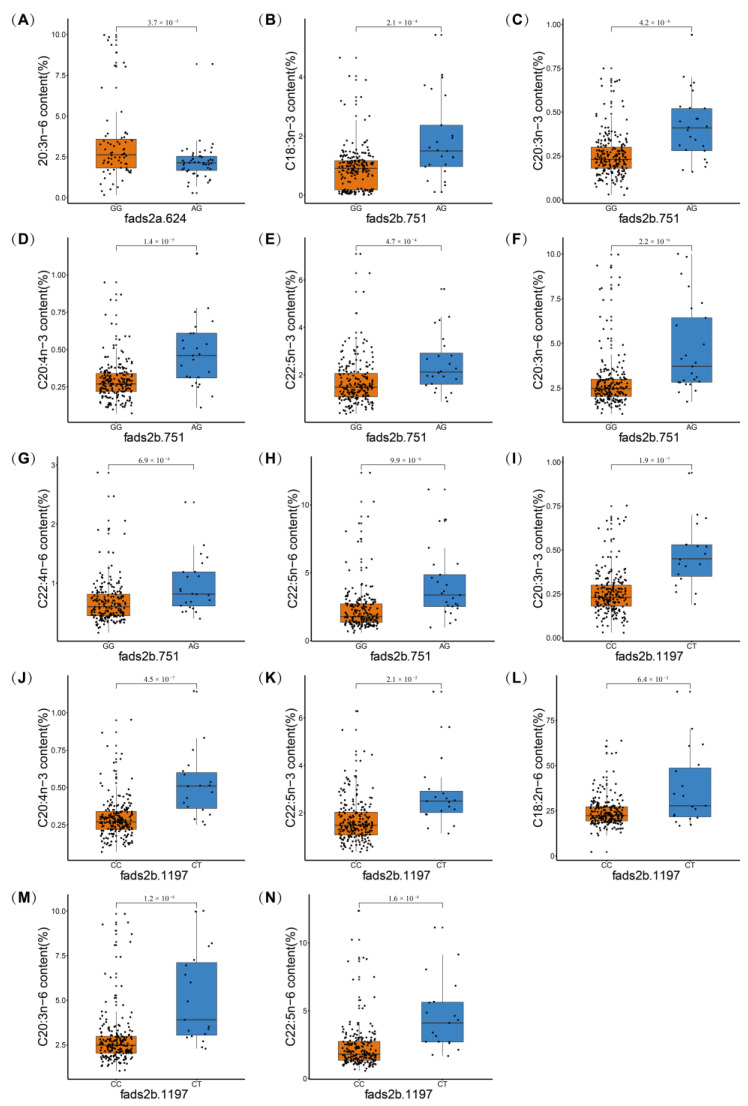
Box plots show significant PUFA content differences between two genotypes in one locus. **A**: fads2a.624; **B**–**H**: fads2b.751; and **I**–**N**: fads2b.1197. The correlated *p* value calculated with ANOVA indicates a significant difference between two groups.

Out of five *fads2a* cSNPs, only one synonymous cSNP (fads2a.624) was found to be significantly associated with the content of C20:3n-6 by both MLM (*p* value of 0.025) and ANOVA (corrected *p* value of 0.0037, Table 4, and Figure 3A). The homozygous genotype (GG) of this locus was associated with higher C20:3n-6 content than the heterozygous genotype (AG), with a fold change of 1.50. 

Among 11 *fads2b* cSNPs, two cSNPs were significantly associated with the contents of multiple PUFAs by both MLM and ANOVA. One non-synonymous cSNP (fads2b.751), resulting in the conversion of valine to isoleucine, was associated with the contents of four n-3 PUFAs (C18:3n-3, C20:3n-3, C20:4n-3, and C22:5n-3) and three n-6 PUFAs (C20:3n-6, C22:4n-6, and C22:5n-6). In this locus, two genotypes including the homozygous genotype (GG) and the heterozygous genotype (AG) were observed while the homozygous genotype of AA was not identified. Interestingly, in all observed associated PUFAs, the individuals with the heterozygous genotype had significantly higher contents than those with the homozygous genotype (Table 4, Figure 3B–H). The fold changes of mean PUFA contents between two groups ranged from 1.4 to 2.1. 

The other cSNP (fads2b.1197) was synonymous and associated with the contents of three n-3 PUFAs (C20:3n-3, C20:4n-3, and C22:5n-3) and three n-6 PUFAs (C18:2n-6, C20:3n-6, and C22:5n-6). In this locus, two genotypes including the homozygous genotype (CC) and the heterozygous genotype (CT) were observed while the homozygous genotype of TT was not identified. Likewise, in all observed associated PUFAs, the individuals with the heterozygous genotype had significant higher contents than those with the homozygous genotype (Table 4, Figure 3I–N). The fold changes of mean PUFA contents between two groups ranged from 1.5 to 2.0.

**Figure 4:**
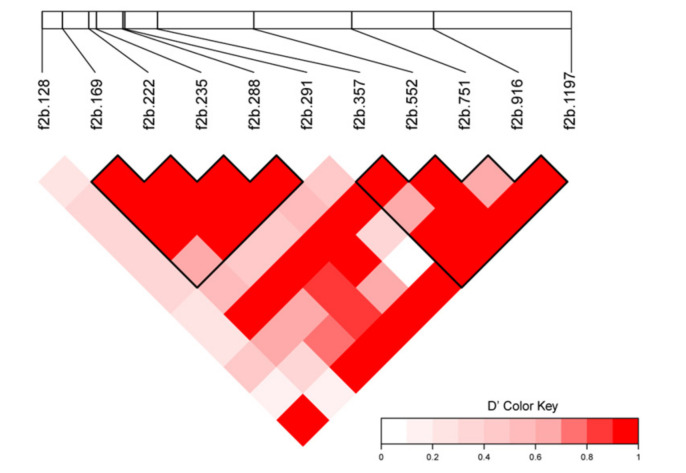
Two haplotype blocks in the *fads2b* CDS. The red color represented tight linkage.

The contents of five PUFAs (C20:3n-3, C20:4n-3, C22:5n-3, C20:3n-6, and C22:5n-6) were associated with both cSNPs. Two haplotype blocks were identified in the *fads2b* CDS (Figure 4). The fads2b.751 and fads2b.1197 were located in the second block, possibly explaining the co-associations of these two cSNPs with five PUFAs contents. However, in these five PUFAs, the non-synonymous cSNPs explained more phenotypic variations than the synonymous cSNP, suggesting that the former was the major effect genotype. 

## 4. Discussion

The limited availability of PUFAs derived from fish represents the critical bottleneck in food production systems, one that numerous research institutions and aqua-feed companies in this field are trying to overcome. Increasing PUFA in fish may have important impacts on human heath, especially in a context where the traditional sources of PUFA, wild fish, and fish oils, are limited by the stagnation of fisheries. Although producing aquaculture fish with more PUFA could be achieved by feeding different fish ingredients [41,42], this goal could be minimized by select-bred fish to be capable of producing endogenous PUFAs more effectively. 

The *fads2* gene is a rate-limiting enzyme catalyzing the desaturation of PUCAs, and the overexpression of salmon *fads2* gene in zebrafish could improve PUFA contents [43]. Two *fads2* genes were identified in the common carp and had differential expression patterns during the development from embryo to larval [23]. Our study intends to find SNPs linked to the PUFA contents in common carp, using a candidate gene approach, by searching associations between polymorphisms in the coding region of *fads2a*/*fads2b* and the PUFA contents. The PUFA content diversities in common carp suggest the possible existence of SNPs in *fads2a*/*fads2b*. Since the genic regions of *fads2a* and *fads2b* were longer than 11,500 bp [23], it is laborious to amplify the whole genic regions. Therefore, we investigated the presence of SNPs in the CDS regions of *fads2a* and *fads2b*. The lower amplification efficiency of *fads2a* than *fads2b* was possibly due to the low gene expression of *fads2a* in liver. 

Although under long-term selection (both natural and artificial) common carp acquired genetic diversity and phenotypic diversity, the three varieties studied had no significant differences in the genetic diversities of these two genes and the PUFA content diversities. Three varieties of common carp were grouped into one population to increase the sample size, the genetic diversities of *fads2a* and *fads2b*, and the PUFA content diversities. It is still unknown which gene makes more contributions to the PUFA biosynthesis. More associated PUFAs and higher explained percentage of phenotypic variations in *fads2b* than *fads2a* suggest that *fads2b* might be the major effect gene for PUFA biosynthesis. More functional studies on these two genes are required for further validation [12,13,15,16]. 

The non-synonymous *fads2b* cSNP might improve desaturase efficiency on the n-3 and n-6 substrates, leading to higher PUFA contents. We also observed one synonymous cSNP associated with the PUFA contents. One possible reason is the tight linkage between the non-synonymous cSNP and the synonymous cSNP, and the synonymous cSNP is a possible biomarker representing the non-synonymous cSNP. The other possible reason is that the synonymous cSNP might affect the DNA methylation level of *fads2b*. In humans, the DNA methylation level in the *fads*1/2/3 gene cluster was linked with genetic variants and desaturase activities and thus affected *fads2* gene expression [44,45]. We predicted two potential CpG islands (from 157 to 731 bp, and from 1015 to 1237 bp) on the *fads2b* CDS region using the method described by Gardiner-Garden and Frommer (1987) with the parameters of GC content over 50% and of the observed/expected ratio over 60% [46]. The synonymous cSNP of fads2b.1197 is located on the second CpG island. Whether this cSNP affects the *fads2b* DNA methylation level and then its expression requires future study. 

We observed the eterozygote advantage with the PUFA contents. The heterozygote advantage was widely observed in the economic traits including the growth rates [47]. In two cSNPs, fads2b.751 and fads2b.1197, the heterozygous genotypes were associated with higher PUFA contents than the homozygous genotypes, suggesting that the heterozygosity of *fads2b* might increase the desaturase activity. Steer et al. [48] reported heterozygotes of *fads2* in humans to be increasingly capable of synthesizing docosahexaenoic acid from alpha-linolenic acid, thereby suggesting that heterozygotes can achieve nutritional adequacy. 

We identified cSNPs in *fads2a* and *fads2b* associated with the PUFA contents. These cSNPs will be applied into future marker-assisted selective breeding of common carp to improve PUFA contents. The question of whether there exist polymorphisms in the promoters, miRNA binding sites, and other functional elements of *fads2a* and *fads2b* will be studied in the future. 

## 5. Conclusions

We detected many polymorphisms in the coding regions of *fads2a* and *fads2b* in common carp and found higher polymorphisms in *fads2b* than *fads2a*. We identified the contents of 12 PUFAs in common carp muscle, which exhibited diversities among individuals. The association studies between the polymorphisms of these two genes and the PUFA contents in common carp identified three cSNPs linked to the PUFA contents. The gene *fads2b* might be the major gene responding for common carp PUFA contents, and fads.751 might be the main effect SNP. Taken together, our findings highlight the importance of polymorphisms in *fads2a* and *fads2b*, critical to the biosynthesis of n-6 and n-3 PUFA levels in common carp. These cSNPs would be potential markers for future selection to improve PUFA contents in common carp.

## Figures and Tables

**Table 1:** The primers used for amplification of the CDS regions of *fads2a* and *fads2b*.

Gene	Primer Sequence (5′-3′)	Accession No.	Tm	Amplification Length (bp)
*fads2a*	F: CAGAGTTTGATCAGTTATGGGCG	MK852165	56 °C	1348
	R: TTTGTTGAGGTACGCATCCAGC			
*fads2b*	F: ATGGGTGGCGGAGGACAGCAG	MK852166	62 °C	1324
	R: GTACGCATCCAGCCAGATTTCTCC			

**Table 2:** Genetic diversities of cSNPs in *fads2a* and *fads2b*.

dbSNP	Position/Exon	Ref/Alt	Ho	He	PIC	MAF	Genotype	Frequencies of Genotypes	Amino Acid Change
Fads2a.288	288/2	G/T	0.1	0.09	0.09	0.05	GG	0.9	
							AG	0.1	
Fads2a.408	408/3	T/C	0.26	0.25	0.22	0.15	TT	0.72	
							CC	0.02	
							CT	0.26	
Fads2a.502	502/3	A/G	0.51	0.4	0.32	0.28	AA	0.46	M-L
							CC	0.03	
							AC	0.51	
Fads2a.561	561/4	C/A	0.3	0.29	0.25	0.18	GG	0.67	
							CC	0.03	
							CG	0.3	
Fads2a.624	624/5	G/A	0.43	0.32	0.27	0.2	GG	0.55	
							AA	0.02	
							AG	0.43	
Fads2b.128	128/1	T/G	0.19	0.17	0.15	0.09	TT	0.81	V-G
							GT	0.19	
Fads2b.169	169/1	C/A	0.3	0.33	0.28	0.21	CC	0.64	Y-H
							AA	0.06	
							AC	0.3	
Fads2b.222	222/2	G/A	0.15	0.45	0.35	0.33	AA	0.59	
							GG	0.26	
							AG	0.15	
Fads2b.235	235/2	A/C	0.16	0.44	0.34	0.33	CC	0.59	
							AA	0.25	
							AC	0.16	
Fads2b.288	288/2	A/G	0.34	0.5	0.37	0.47	AA	0.36	L-M
							GG	0.3	
							AG	0.34	
Fads2b.291	291/2	T/C	0.38	0.48	0.36	0.4	TT	0.41	I-L
							CC	0.21	
							CT	0.38	
Fads2b.357	357/3	T/C	0.35	0.49	0.37	0.45	TT	0.37	
							CC	0.28	
							CT	0.35	
Fads2b.552	552/4	T/C	0.13	0.13	0.12	0.07	TT	0.86	H-Q
							CC	0.01	
							CT	0.13	
Fads2b.751	751/6	G/A	0.1	0.1	0.1	0.05	GG	0.9	V-I
							AG	0.1	
Fads2b.916	916/8	T/C	0.02	0.34	0.28	0.22	TT	0.77	
							CC	0.21	
							CT	0.02	
Fads2b.1197	1197/11	C/T	0.08	0.07	0.07	0.04	CC	0.92	
							CT	0.08	

**Table 3:** Proportions of PUFA composition.

n-3 PUFA	Composition (%)	n-6 PUFA	Composition (%)
18:3n-3	0.98 ± 0.88	18:2n-6	24.33 ± 11.03
18:4n-3	0.1 ± 0.19	18:3n-6	6.36 ± 6.75
20:3n-3	0.32 ± 0.48	20:3n-6	2.85 ± 1.74
20:4n-3	0.29 ± 0.15	20:4n-6	8.17 ± 4.96
20:5n-3	3.39 ± 2.21	22:4n-6	0.67 ± 0.39
22:5n-3	1.69 ± 0.99	22:5n-6	2.37 ± 1.84
Total	6.76 ± 3.58	Total	44.68 ± 16.81

All data are presented as the mean ± standard error (SE).

**Table 4:** Association analysis of PUFA contents using MLM and ANOVA.

Trait	SNP	MLM (PCA + K)		ANOVA		
PUFA	ID	*p* Value	Marker R^2^ (%)	Genotype		FDR *p* Value
				MM	Mm	
Fads2a						
C20:3n-6	fads2a.624	2.50 × 10^−2^	4.4	3.26 ± 2.35	2.18 ± 1.12	3.7 × 10^−3^
Fads2b						
C18:3n-3	fads2b.751	1.0 × 10^−2^	5	0.9 ± 0.72	1.85 ± 1.39	2.1 × 10^−4^
C20:3n-3	fads2b.751	2.7 × 10^−6^	13	0.25 ± 0.12	0.42 ± 0.19	4.2 × 10^−6^
C20:4n-3	fads2b.751	3.2 × 10^−5^	11	0.29 ± 0.12	0.47 ± 0.22	1.4 × 10^−5^
C22:5n-3	fads2b.751	2.0 × 10^−5^	11	1.67 ± 0.93	2.47 ± 1.19	4.7 × 10^−4^
C20:3n-3	fads2b.1197	1.7 × 10^−3^	4.3	0.25 ± 0.12	0.47 ± 0.18	1.9 × 10^−7^
C20:4n-3	fads2b.1197	8.5 × 10^−4^	5	0.29 ± 0.13	0.52 ± 0.21	4.5 × 10^−7^
C22:5n-3	fads2b.1197	4.7 × 10^−4^	5	1.66 ± 0.88	2.81 ± 1.43	2.1 × 10^−6^
C20:3n-6	fads2b.751	2.7 × 10^−5^	11	2.78 ± 1.44	4.69 ± 2.47	2.2 × 10^−6^
C22:4n-6	Fads2b.751	3.1 × 10^−4^	7	0.68 ± 0.36	0.95 ± 0.45	6.9 × 10^−4^
C22:5n-6	Fads2b.751	2.7 × 10^−3^	9	2.28 ± 1.59	4.08 ± 2.5	9.9 × 10^−6^
C18:2n-6	Fads2b.1197	2.5 × 10^−3^	4	24.19 ± 7.83	37.28 ± 20.34	6.4 × 10^−3^
C20:3n-6	Fads2b.1197	2.0 × 10^−3^	4	2.79 ± 1.44	5.17 ± 2.52	1.2 × 10^−6^
C22:5n-6	Fads2b.1197	1.2 × 10^−6^	3	2.29 ± 1.58	4.59 ± 2.55	1.6 × 10^−6^

Marker R^2^: percentage of phenotypic variation explained by markers in MLM (PCA + K) method; K: Kinship matrix; M: major allele; m: minor allele. The data are presented as the mean ± SE represented the PUFA content in one genotype group.

## Data Availability

The data presented in this study are available in supplementary material.

## Supplementary Materials

The following are available online at https://www.mdpi.com/article/10.3390/ani11061780/s1, Table S1: The genotypes of fads2a and fads2b, Table S2: The contents of 12 PUFAs in 269 samples, Figure S1: The images of three varieties of common carp used in this study, Figure S2: Population structures using the genotypes of fads2a and fads2b, Figure S3: PCA plot clustering three common carp varieties with the contents of 12 PUFAs, Figure S4: Comparing the content distributions of 12 PUFAs with the normal distribution.
