# Supplementary material for: Association between the Polymorphisms of fads2a and fads2b and Poly-Unsaturated Fatty Acids in Common Carp (Cyprinus carpio)
Source: Animals (Basel). 2021 Jun 15;11(6):1780. doi: 10.3390/ani11061780 (PMC8232129; doi:10.3390/ani11061780)
Supplement: Supplementary file 1 [file animals-11-01780-s001.zip › animals-1224639-supplementary.pdf]

Supplementary Tables

Table S1. The genotypes of *fads2a* and *fads2b*

(individual excel file)

Table S2. The contents of 12 PUFAs in 269 samples

(individual excel file)

Supplementary Figures.

Figure S1. The images of three varieties of common carp used in this study. Common carp var. Jian (A), Common carp var. Furui (B), and Common carp var. Huanghe (C).

(A)

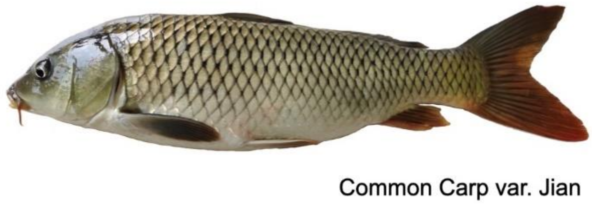

(B)

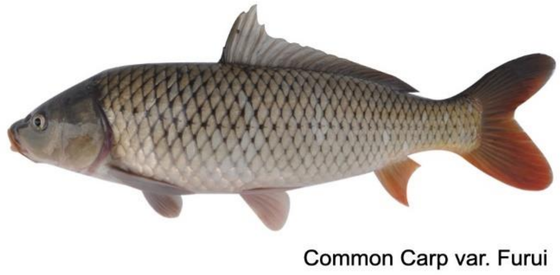

(C)

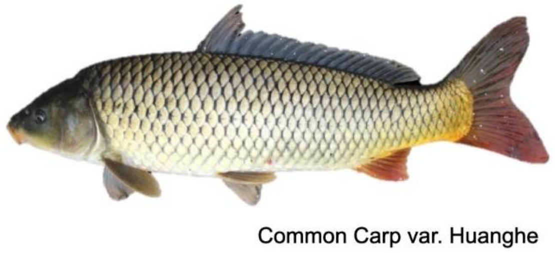

Figure S2. Population structures using the genotypes of *fads2a* and *fads2b*, respectively.

(A)

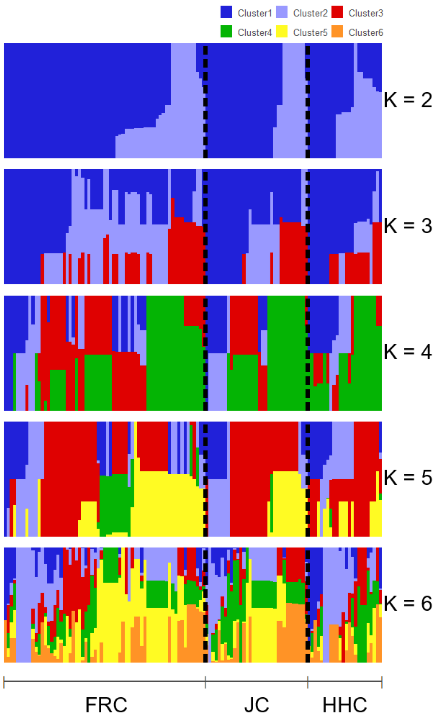

(B)

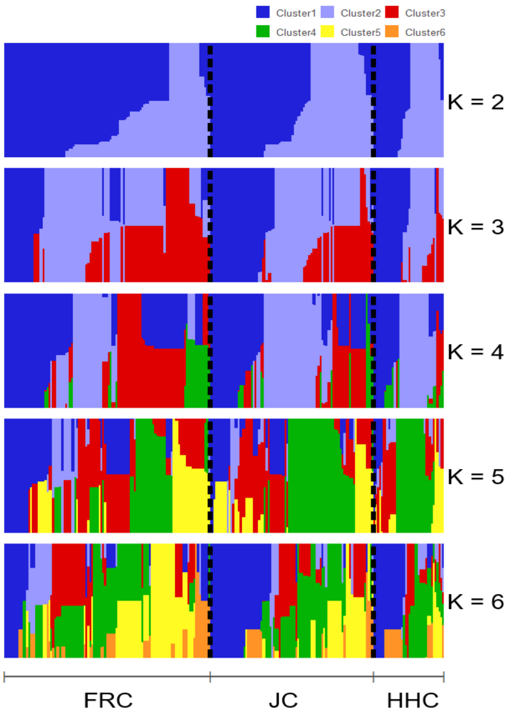

Figure S3. PCA plot clustering three common carp varieties with the contents of 12 PUFAs.

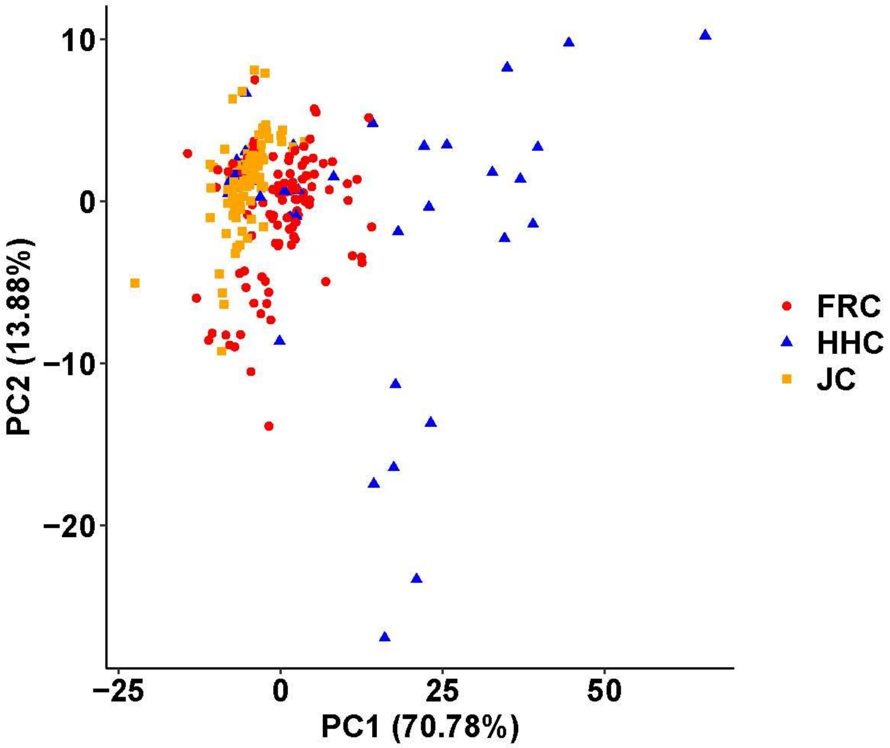

Figure S4. Comparing the content distributions of 12 PUFAs with the normal distribution

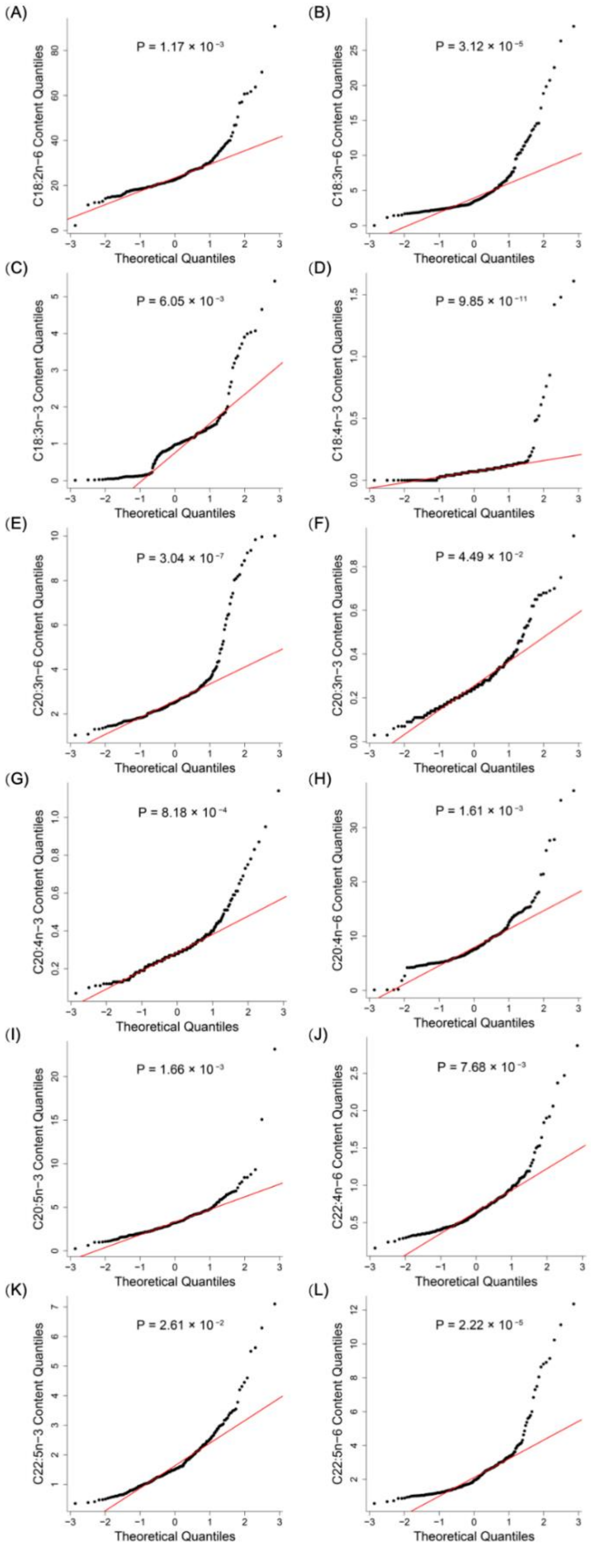

The Kolmogorov-Smirnov test p value listed in the figure showed the PUFA content was not in the normal distribution.
